# Fungus Among Us: An 8-Year Retrospective Study of Fungal Infections at a Tertiary-Care Hospital in Bucharest, Romania

**DOI:** 10.3390/pathogens14101061

**Published:** 2025-10-20

**Authors:** Alina Maria Borcan, Mihaela-Cristina Olariu, Teodora Gabriela Anghel, Elena Rotaru, Bianca Secuiu, Madalina Simoiu, Narcis Copca, Dragos Cretoiu, Laura Georgiana Caravia

**Affiliations:** 1 Faculty of Medicine, The University of Medicine and Pharmacy “Carol Davila”, Dionisie Lupu Street, No. 37, 050474 Bucharest, Romania; alina.borcan@umfcd.ro (A.M.B.); mihaela.olariu@umfcd.ro (M.-C.O.); teodoraanghel21@gmail.com (T.G.A.); rotaru.hellen@gmail.com (E.R.); narcis.copca@umfcd.ro (N.C.); dragos@cretoiu.ro (D.C.); laura.caravia@umfcd.ro (L.G.C.); 2 The National Institute of Infectious Diseases “Prof. Dr. Matei Balș”, Doctor Grozovici Street, No. 1, 021105 Bucharest, Romania; 3 St. Mary Clinical Hospital, Department of General Surgery, Ion Mihalache Boulevard, No 37-39, 011172 Bucharest, Romania; 4 The National Institute of Mother and Child Health “Alessandrescu-Rusescu”, Materno-Fetal Medicine Excellence Center, Lacul Tei Boulevard, No 120, 20382 Bucharest, Romania

**Keywords:** antifungal resistance, retrospective study, *Candidozyma auris*, candidemia, *Aspergillus*, urinary tract infection, fungi, fluconazole

## Abstract

Despite the devastating consequences of fungal disease, research struggles to catch up to present needs. This study aims to give a broad perspective on the situation, investigating patterns and distribution of fungal pathogens and monitoring trends of resistance to antifungal drugs, over an 8-year timeframe, at the National Institute of Infectious Diseases “Prof. Dr. Matei Balș” in Bucharest, Romania. Samples were inoculated on Sabouraud or Brilliance Candida Agar media; strains were identified using MALDI-TOF MS; and antifungal sensitivity testing was performed using E-Tests strips, VITEK2 Compact and MICRONAUT-AM automatic systems. *Candida albicans*, accounting for 42% of the positive samples, was the most common pathogen observed, with only 17% of the isolates being susceptible to all antifungals tested, while it was also predominant and deadly in the ICU. The emerging *Candidozyma auris*, found in 8% of the candidoses, exhibited a fluconazole resistance rate of 96.3%. Of the *Aspergillus fumigatus* strains, 35.7% showed resistance to azoles, and 25% to amphotericin B. In the ICU, more than half of *A. flavus*-, *A. fumigatus*- or *A. niger*-related cases culminated in death. Antifungal resistance is not to be treated lightly, as it is still a complex and dynamic threat, with devastating consequences.

## 1. Introduction

Fungi are a distinct group of eukaryotes that are set apart from plants and animals, with enormous influence in nature. They can occur as uni- or multicellular structures and are characterized as heterotrophic, requiring preformed organic carbon compounds for their nutrients [1]. These microorganisms are often characterized as being opportunistic, especially affecting individuals with a weakened immune system, whether this weakness is due to malignancy, HIV, tuberculosis, diabetes mellitus or chronic obstructive pulmonary disease; individuals who receive immunosuppressant treatments; or patients from intensive care units who receive broad-spectrum antibiotics. *Candida* spp., *Aspergillus* spp. and *Cryptococcus neoformans* are just some examples, with more than 400 species being included here. Less than 100 species have the status of human pathogens frequently involved in invasive mycoses, such as *Blastomyces dermatitidis*, *Histoplasma capsulatum* and *Coccidioidis immitis* [2,3].

In 2022, the World Health Organization (WHO) classified pathogenic fungi in groups based on the risk imposed, recognizing *Candida albicans*, *Cryptococcus neoformans*, *Aspergillus fumigatus* and *Candidozyma auris* (formerly known as *Candida auris*) as the most critical fungi that cause infections in humans. First described nearly 15 years ago, *Cz. auris* is now reported across all continents except Antarctica, constituting a pressing public health challenge due to widespread antifungal resistance and elevated mortality, with over 1000 publications addressing this emerging pathogen, of which roughly 15% include genomic analyses [4]. The reclassification of *Candida auris* into *Candidozyma auris* was proposed in 2023, with the formal new name being applied in 2024 [5]. Other species of *Candida*, such as *C. glabrata*, *C. tropicalis* and *C. parapsilosis*, joined *Histoplasma* spp. and *Fusarium* spp. in the high-risk category [6]. Overall, *Candida* spp. have been the most incriminated pathogen in fungal infections, with more than 15 species being associated with diseases in humans, but it is important to keep in mind that non-*Candida* spp. infections are continuously on the rise [7]. Fungal disease can present as superficial infections of the skin and nails (most common), as mucosal infections of the oral and genital tract or as invasive infections, with a reduced incidence but with concerning high mortality rates. Studies show that the number of fatal invasive fungal infections is comparable with the mortality associated with tuberculosis or malaria [8].

Infections caused by these organisms, oftentimes overlooked, represent a major global health issue, as these pathogens are becoming increasingly frequent and, most importantly, tend to be resistant to treatments. That happens via intrinsic mechanisms, such as reduced drug–target binding, elevated efflux pump activity and distinctive cell wall or membrane composition, as well as through acquired mechanisms, including genetic mutations, gene duplication, transposon insertions, aneuploidy and loss of heterozygosity. There is also the notion of antifungal tolerance, defined by subpopulations of fungal cells that are capable of persisting and growing despite high drug concentrations, that further complicates the treatment plan. Another mechanism by which fungal pathogens evade antimycotic drugs is through biofilm formation, which commonly develops on abiotic surfaces, yet biofilms are likewise capable of forming on biotic surfaces, such as mucosal epithelial linings [9]. Biofilms offer physical protection against antimycotic drugs through extracellular matrix production, while the cells within present a constitutive upregulation of drug-efflux pumps and altered metabolic states [10]. There is ongoing research on antimycotic natural agents that target biofilms, with promising candidates against various pathological fungi being yielded. Among these, essential oils included in nanosystems showed antibiofilm properties against not only *C. albicans* but also *C. glabrata* and *C. tropicalis*, yet they were not as successful against *Candidozyma auris*. However, the protein NFAP2 and the highly functionalized polycyclic compound turbinmicin show notable potential, as they seem to completely disrupt extracellular vesicle delivery during *Cz. auris* biofilm formation, thereby impairing a key virulence mechanism. Similarly, rubiginosin C, isolated from the ascomycete *Hypoxylon rubiginosum*, safely inhibited the formation of biofilms, pseudohyphae and hyphae in both *C. albicans* and *Cz. auris*. In parallel, secondary metabolites from an *Aspergillus* strain collected near Waikiki Beach in Hawaii led to the identification of a novel prenylated indole alkaloid named waikialoid A that successfully suppressed *C. albicans* biofilm formation, without exerting cytotoxicity toward human cells at certain concentrations. Additionally, propolis derived from Brazil has demonstrated antimycotic efficacy against *Fusarium* spp. [11,12,13].

In Romania, an epidemiological study revealed that in 2016, an estimated 2.23% of the population was affected by a serious form of fungal disease [14]. A 6-year study (2009–2014) evaluating the trend of species distribution and antifungal susceptibility patterns of invasive strains of *Candida* spp., conducted at a university hospital located in the central part of the country, showed that *C. parapsilosis* was the most commonly isolated pathogen, with a fluconazole resistance of 5%, followed by *C. albicans*, with no fluconazole and echinocandin resistance detected [15]. It is quite noticeable that available data assessing the fungal burden in Romania is either outdated or limited in scope, focusing on specific infection types, fungal species or clinical cases. Therefore, our study aims to provide a more comprehensive and broader overview of the current situation, filling the gaps and offering an updated view of the national fungal landscape.

## 2. Materials and Methods

This study is based on retrospective data collection conducted from 1 January 2017 to 31 December 2024, covering a period of 8 years, obtained from records in the Microbiology Laboratory of the National Institute of Infectious Diseases “Matei Balș”, a mono-disciplinary tertiary-care hospital in Bucharest, Romania. The total number of yeast strains added up to 812, from both inpatients and outpatients of all ages, from our hospital wards or the Intensive Care Unit (ICU). Upon hospital admission, informed consent was secured from each participant or their legal guardian, with data subsequently being anonymized. Fungi were obtained from a range of various samples: urine, cerebrospinal fluid, sputum, stool samples, blood, pharyngeal and nasal swabs, lingual scrapings, bronchial and tracheal aspirates, wound samples, synovial fluid, and other secretions that have a dedicated sector called “Diverse secretions”. This includes the following: vaginal, peritoneal, otic, pericardial and conjunctival secretions; catheter samples; and ascitic fluid. We also examined samples from the ICU, collected on a tampon from axillary and inguinal areas, for *Candidozyma auris* colonization. As the records span from 2017 to 2024, we will present *Candidozyma auris* within the *Candida* section, to maintain consistency with the data collected, while referring to the updated nomenclature, using the current name throughout the text, in order to reflect, support and acknowledge the ongoing implementation of this recent taxonomic change. As this is still in the process of being implemented in clinical and research settings, this approach is clearer for the reader and more comparable with studies up until 2024.

All samples were collected in sterile containers, before any antifungal treatment was administered, following rigorous protocols and inoculated on Sabouraud media (SABGC, BioMereux, Salt Lake City, UT, USA), containing chloramphenicol and gentamicin, with the exception of lingual scraping samples and axillary and inguinal tampons that were inoculated on Brilliance Candida Agar (BioMereux, Salt Lake City, UT, USA), a chromogenic medium for easier identification of *Candida* spp. The procedure continued with an incubation period of 18 ± 2 h at 35 ± 2 °C, after which plates were stored at room temperature for 4 additional days and assessed daily until reaching the endpoint of 5 days (120 h) post-incubation. Direct microscopic identification was performed for cerebrospinal fluid samples using India Ink to identify *Cryptococcus neoformans.* All 812 strains were identified using Matrix-Assisted Laser Desorption Ionization Time-of-Flight Mass Spectrometry (MALDI-TOF MS, Bruker, Billerica, MA, USA). Fungal spectra were analyzed using the Biotyper^®^ software version 3.1 (Bruker Daltonik GmbH, Bremen, Germany). Antifungal sensitivity testing was performed using E-Tests strips, VITEK2 Compact (BioMereux, Salt Lake City, UT, USA) and MICRONAUT-AM (Bruker, Billerica, MA, USA) automatic systems. Results were interpreted according to the EUCAST official clinical breakpoints. Organization and analysis of quantitative data were conducted using Microsoft Excel and Microsoft Word (2024 version).

## 3. Results

### 3.1. General Distribution of Fungal Strains

**Figure 1:**
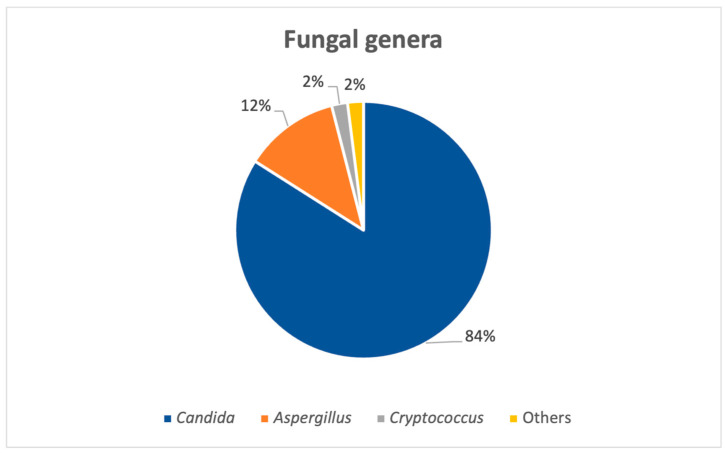
General distribution of fungal genera.

A total of 812 fungal strains from 734 patients were isolated in an 8-year period, between 2017 and 2024, from both pediatric (N = 49) and adult (N = 685) patients. The number of positive fungal samples was higher after the COVID-19 pandemic (from 2021 to 2024) versus the total number for the pandemic and pre-pandemic years (2017–2020) (N = 502 for post-pandemic; N = 310 pre-pandemic and pandemic years). Overall, the most common fungal genus isolated was *Candida* spp., as seen in Figure 1, adding up to roughly 84% (N = 682) of the strains, followed by *Aspergillus* species.

### 3.2. Distribution of Fungi Among Patient Samples

**Figure 2:**
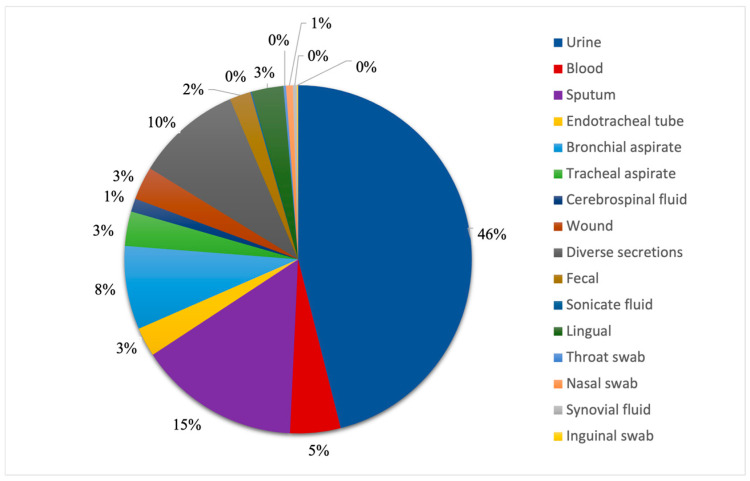
Distribution of positive fungal patient samples.

We looked at all patient samples positive for fungal infections, and we observed that almost half of them were from urine samples, which accounted for 46% (N = 374) of the total specimens, as seen in Figure 2. The second most common sample was sputum, which represented 15% of the total patient samples (N = 122). It is worth noting that the combined number of sputum, endotracheal tube, bronchial and tracheal aspirate samples, which represent all respiratory system specimens, accounts for 29% (N = 233) of all samples in this study. Diverse secretions made up 10% of them (N = 80), being ranked third, from which the most common secretion was otic, adding up to 16 samples. The lowest numbers of samples were represented by synovial fluids (N = 3) and inguinal swabs (N = 1).

**Figure 3:**
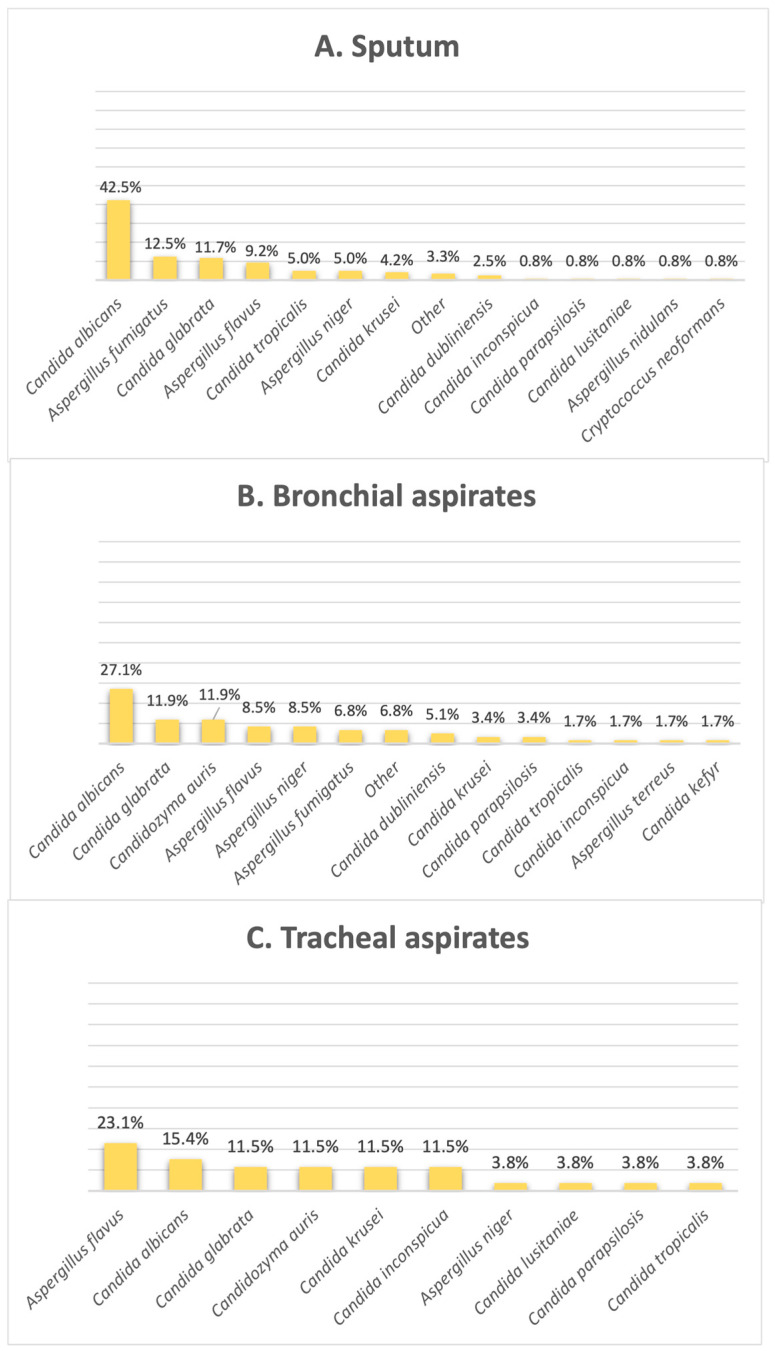
Distribution of fungi in the respiratory tract: (**A**) sputum, (**B**) bronchial aspirates, (**C**) tracheal aspirates.

Figure 3 shows that the most common fungi species found in sputum and bronchial aspirates was *Candida albicans* (42.5% in sputum, 27% in bronchial aspirates); however, when it comes to tracheal aspirates, this is no longer available, with this species falling off in second place. As illustrated by Figure 3, *Aspergillus flavus* is incriminated as the most frequent fungal isolate found in tracheal aspirates, making up 23% of this clinical sample type. *Candidozyma auris* shares the second position with *C. glabrata* in bronchial aspirates, found in 11.8% of the results, as well as the third position in tracheal aspirates, making up 11.5% of the samples in this category.

**Figure 4:**
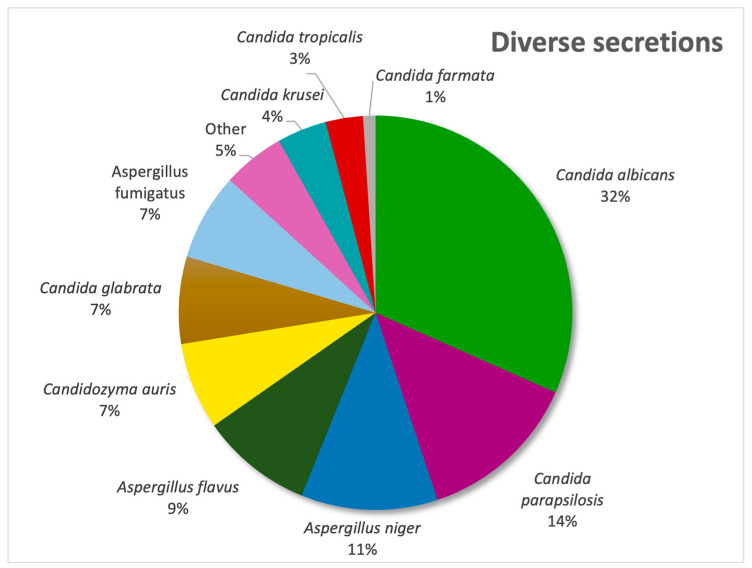
Distribution of fungal pathogens in diverse secretions.

**Figure 5:**
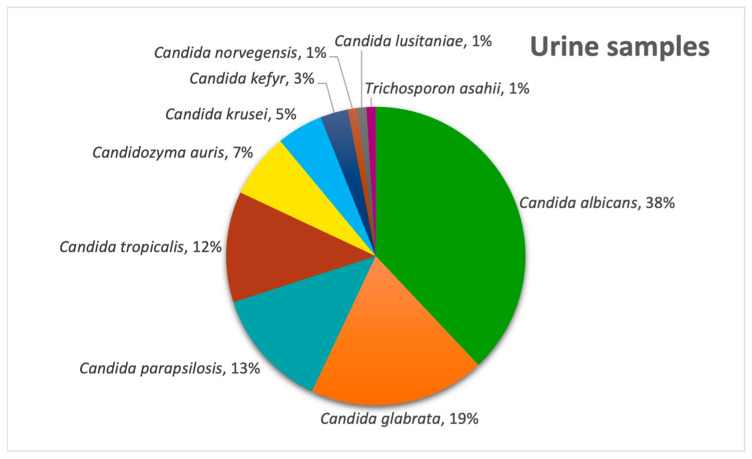
Distribution of fungal pathogens in urine samples.

In our diverse secretion sector, *Candida albicans* was the most frequently isolated pathogen (31%), followed by *Candida parapsilosis* (13%) and *Aspergillus niger* (11%), as evidenced by Figure 4. In urine samples, *C. albicans* remained predominant (38%), with *C. glabrata* second (19%) and *Candida parapsilosis* being third (13%) (Figure 5) and with the only non-*Candida* isolate being *Trichosporon asahii*.

In stool cultures, from the total of 15 positives in our database, 8 cases involved *Candida glabrata*, followed by 6 cases of *Candida albicans* and 1 case of *Candida dubliniensis*. In cerebrospinal fluid samples, analysis found that *Cryptococcus neoformans* was positive in 9 out of 10 cases, with the only remaining positive case being attributed to *Candida albicans*.

**Figure 6:**
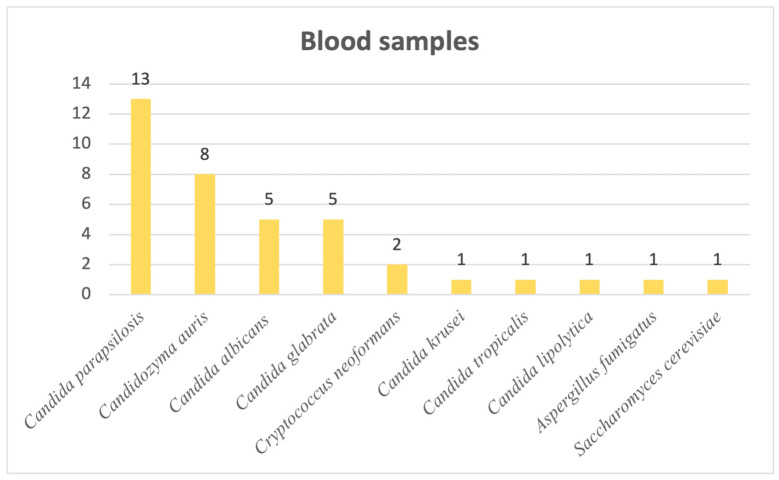
Distribution of fungal pathogens in blood samples.

Last but not least, when it comes to blood samples, we observed the following distribution: 34 out of 38 cases of systemic mycoses were candidoses, with 2 cases of infection caused by *Cryptococcus neoformans* and, lastly, 1 caused by *Aspergillus fumigatus* and 1 by *Saccharomyces cerevisiae.* Most frequently responsible for systemic candidoses was *Candida parapsilosis*, followed by the newly emerging *Candidozyma auris*. Other *Candida*, *Cryptococcus*, *Aspergillus* and *Saccharomyces* species are visible in Figure 6.

### 3.3. Intensive Care Unit

**Figure 7:**
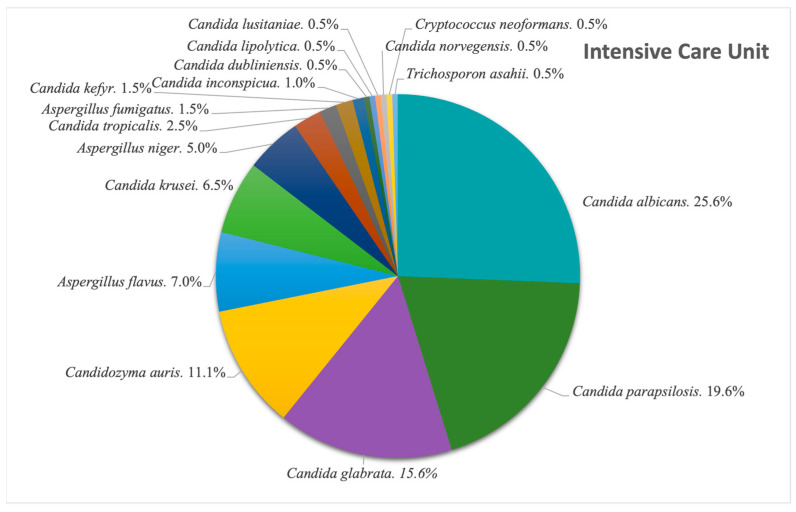
Distribution of fungal pathogens in the Intensive Care Unit (ICU).

We looked at the situation in our ICU, and observed, as seen in Figure 7, the following: Among the 812 fungal isolates extracted in the 8-year timeframe, 199 were from the ICU (24.5%), of which *Candida albicans* was the most common species found (25.6%; N = 51). This is followed by *Candida parapsilosis* (19.6%; N = 39) and *Candida glabrata* (15.6%; N = 31). The newly emerging *Cz. auris* made its way to the fourth place, adding up to 22 cases (11.1%) of the ICU isolates. The next most common genus after *Candida* spp. was *Aspergillus* spp., with *A. flavus* accounting for 7% of the isolates, translating into 14 cases.

**Figure 8:**
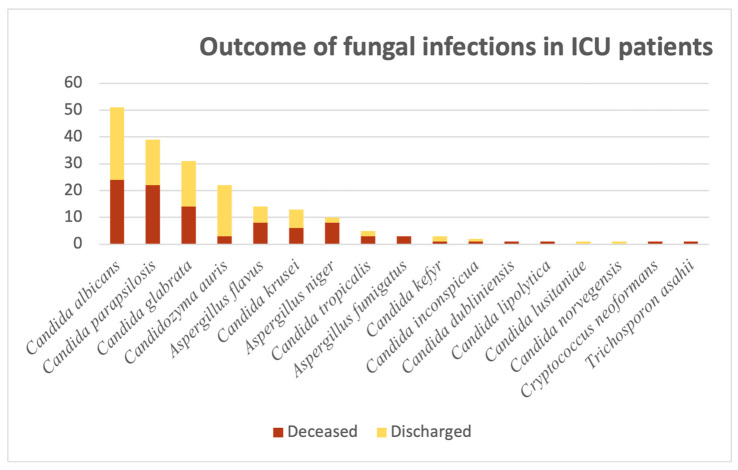
Outcome distribution by fungal etiology in ICU cases.

Given the harsh nature of the ICU, we were interested in having a bird’s-eye view of the patients’ outcomes, divided into “Deceased” and “Discharged”, as illustrated in Figure 8 below. Out of the 51 *C. albicans*-related cases, almost half of them culminated in death (47%; N = 24). For the next *Candida* species, the proportions are of greater concern: Out of 39 cases of *C. parapsilosis*, more than half were associated with the patient’s death (56.4%; N = 22). *C. glabrata*-related infections accounted for 31 cases, out of which 14 had a negative outcome (45.1%). Patients in the ICU with positive *Candidozyma auris* infections had better outcomes: out of the 22 cases, only 3 (13.6%) resulted in death.

The situation for *Aspergillus* spp. cases can be summarized as follows: *A. flavus*-related cases accounted for 14 patients, out of which 8 resulted in death (57.1%); *A. niger* isolates accounted for 10 cases, out of which 8 resulted in death (80%). For *A. fumigatus*-related infections, all three cases resulted in death. Aside from *Candida* and *Aspergillus* species, we encountered one *Cryptococcus neoformans* and one *Trichosporon asahii*-related case, both with a negative outcome.

### 3.4. Candida and Candidozyma Genera

**Figure 9:**
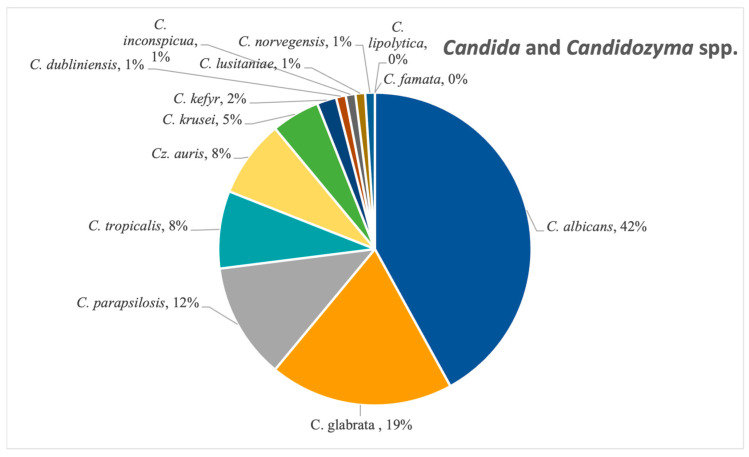
Species distribution of *Candida* and *Candidozyma* species.

*Candida albicans* was the most frequent Candida species isolated, confirmed in 42% of the candidoses (N = 287), followed by *Candida glabrata* (19%, N = 127), as seen in Figure 9. Other species from this genus that were detected in a significant amount were *C. tropicalis*, *C. parapsilosis* and *C. krusei*. The emerging *Candidozyma auris* was also found in 8% (N = 54) of the samples from this category, notably between 2022 and 2024.

**Figure 10:**
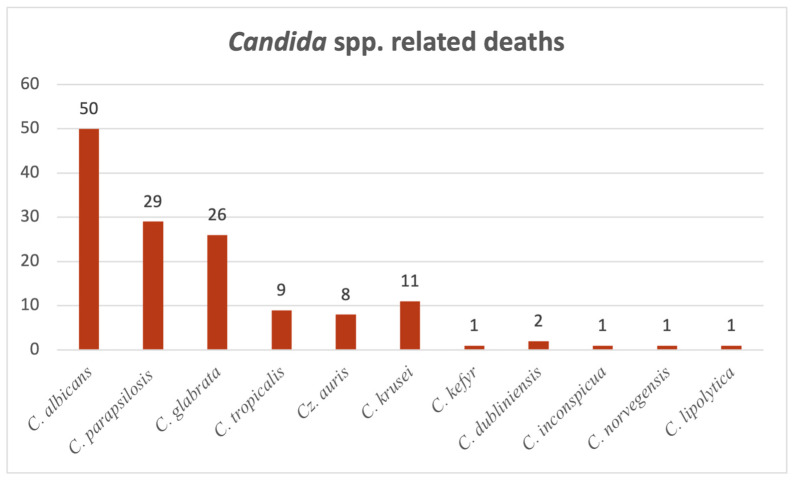
Distribution of deaths by *Candida* species.

Figure 10 presents deaths occurring in patients where *Candida* spp. infections were detected, both in hospital wards and in the ICU. Altogether, 139 patients with documented candidosis subsequently died. Accounting for the majority of them (35.9%; N = 50), *Candida albicans* takes the lead here as well. *Candida parapsilosis*-associated deaths amounted to 29 cases (20.8%) and took the lead in positive blood samples, followed by *Candida glabrata* with 26 cases (18.7%).

**Figure 11:**
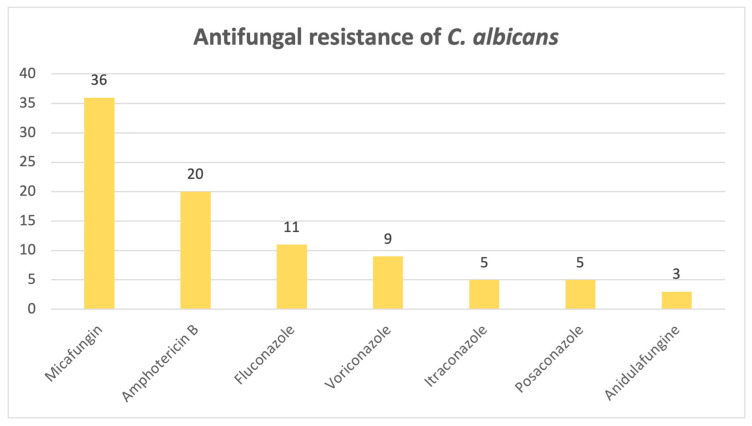
Antifungal resistance of *Candida albicans*.

When it comes to the antifungal resistance patterns of the most common fungi isolated, *Candida albicans*, Figure 11 shows that out of 297 positive cases found, 66 strains exhibited resistance to at least one antifungal drug. Among the resistant strains, most of them were found to be resistant to micafungin (N = 36) and amphotericin B (N = 20).

*Candida krusei* remains susceptible in vitro to voriconazole, while showing a reduced susceptibility to amphotericin B: out of 35 samples, 3 were resistant.

**Figure 12:**
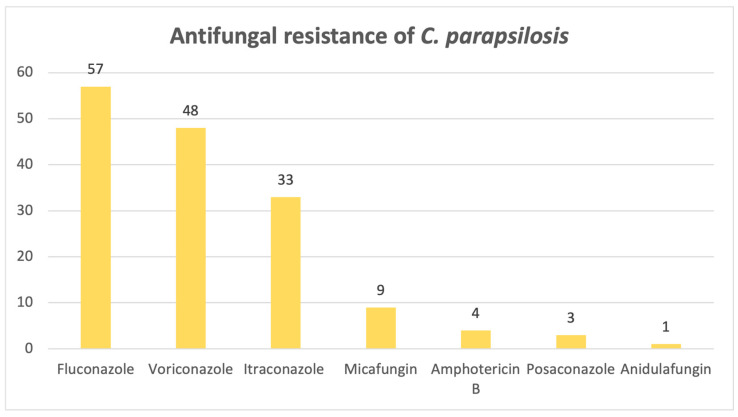
Antifungal resistance of *Candida parapsilosis*.

*Candida parapsilosis* exhibits a significant resistance to azoles, as evidenced below in Figure 12: out of 82 samples, 57 were resistant to fluconazole, 48 to voriconazole and 33 to itraconazole.

**Figure 13:**
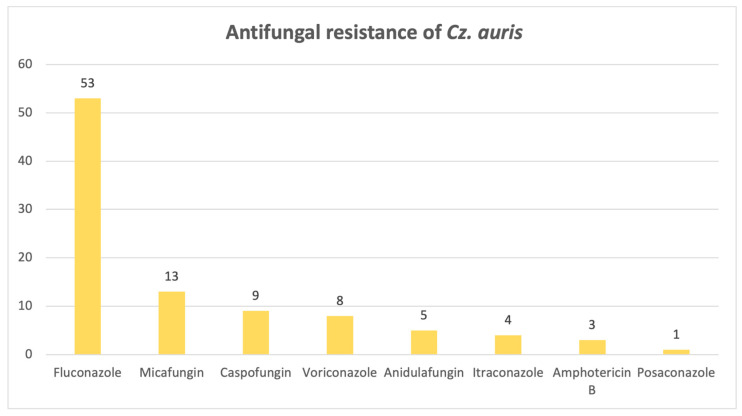
Antifungal resistance of *Candidozyma auris*.

Lastly, Figure 13 illustrates a significant resistance of *Candidozyma auris* to fluconazole: out of 55 strains, 53 are resistant.

### 3.5. Cryptococcus Genus

*Cryptococcus neoformans* is the only species found in our studied samples, mainly from cerebrospinal fluid (N = 9) and blood (N = 2), but also from 1 wound sample and 1 sputum sample, adding up to 13 cases. One patient was from the ICU, and the rest of them were admitted to adult wards. Out of these, the patient from the ICU and two others who were infected with this fungal pathogen had negative outcomes.

Out of the 13 cases, 2 strains were resistant to fluconazole, both extracted from cerebrospinal fluid samples, one of which resulted in the death of the patient.

### 3.6. Aspergillus Genus

**Figure 14:**
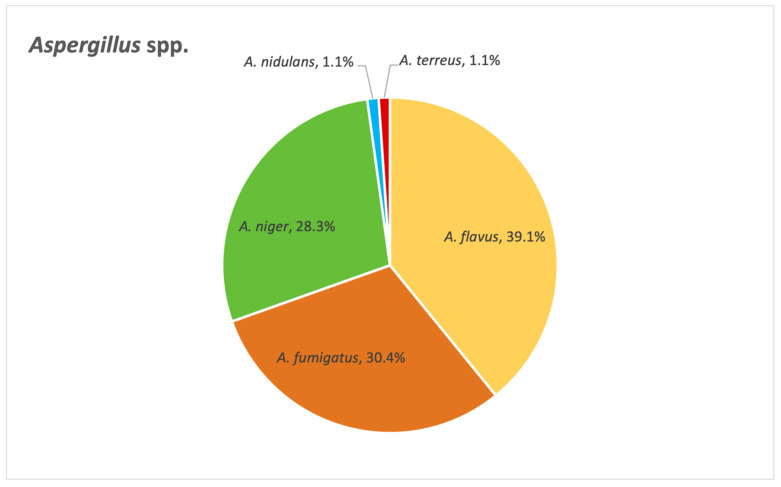
Distribution of *Aspergillus* species.

Figure 14 below depicts the distribution of *Aspergillus* species identified in our study. Among these, *A. flavus*, *A. fumigatus* and *A. niger* emerge as the most frequently encountered species.

Next, we wanted to evaluate the outcome of these cases. Out of 29 cases associated with *A. niger*, 10 had a negative outcome. The situation is slightly better when it comes to *A. flavus:* out of 34 cases, 8 resulted in death. The same can be said about *A. fumigatus* as well: out of 28 cases, 6 progressed to a fatal outcome. As for *Aspergillus terreus*, the only case in our database culminated in death.

Moving forward, we addressed antifungal resistance among the most prevalent species, starting with *Aspergillus flavus*. Out of the 34 cases, 17 strains showed resistance to one or more antifungal drugs. We observed that most of the strains exhibited resistance to amphotericin B (N = 9), followed by fluconazole (N = 8). Other drugs from the azole class, namely posaconazole and voriconazole, were more susceptible, as only two strains were found to be resistant to the first-mentioned drug and just one strain was resistant to voriconazole.

**Figure 15:**
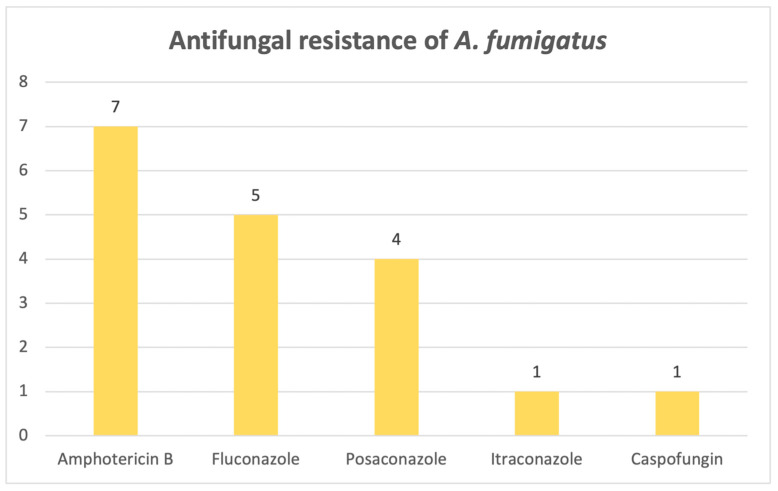
Antifungal resistance of *Aspergillus fumigatus*.

For *Aspergillus fumigatus*, resistance patterns are widespread across more types of classes. Out of 28 identified cases, 13 strains showed resistance to at least one antifungal drug. Figure 15 reveals that the highest resistance corresponded to an antifungal from the polyene class, namely amphotericin B (N = 7), followed by azoles (fluconazole, posaconazole, itraconazole) and, lastly, a drug from the echinocandine class, caspofungin. One strain showed resistance to three antifungal drugs simultaneously (amphotericin B, itraconazole, posaconazole), and it was identified in a wound sample.

*Aspergillus niger* exhibits the lowest resistance rates out of all filamentous fungi: out of 28 cases, 8 showed antifungal resistance, with 6 of the strains being resistant to fluconazole, 1 to amphotericin B and 1 to itraconazole.

## 4. Discussion

Our study demonstrated an upward trend in the number of positive fungal infections, with most positive samples from this 8-year timeframe coming from urine. *Candida albicans* was found to be the most common pathogen observed in our hospital, which is to be expected, as the most common mycosis in developed countries is represented by candidosis [16]. *C. albicans* was also predominant in the ICU, where almost half of the patients who had infections associated with this species culminated in death. Patients in intensive care units are at a particularly high risk of developing fungal infections, whether it is due to invasive procedures, prolonged hospitalization, nosocomial infections or immunosuppression. These factors create a favorable scene for opportunistic fungi, most commonly *Candida* spp., predisposing critically ill patients to fungal colonization and infection [17]. While it is associated with invasive mechanical ventilation, it is difficult to distinguish colonization from true infection, since *Candida*-related ventilator-associated pneumonia is rare [18]. Despite its designation by the WHO as a pathogen not generally associated with antifungal treatment resistance, our laboratory found only a small percentage of the *C. albicans* isolates to be susceptible to all antifungals tested. The highest resistance rates were observed for micafungin, amphotericin B and fluconazole. As for other species, for example, *C. krusei* and *C. parapsilosis*, also ranked as high-risk by the WHO and responsible for most invasive candidoses [16], the highest resistance rate was exhibited against fluconazole. *Candida parapsilosis* was considered susceptible to azoles; however, there has been a progressively growing number of cases associating a resistance to fluconazole without the patient ever receiving this particular treatment before, which further proves the theory that transmission between patients is found to be at fault for the increasing number of resistant cases, therefore posing a serious concern, given that mortality rates may reach up to 78% [19]. According to a surveillance study performed from 1997 to 2016, the general trend was that the number of non-*Candida albicans* species isolates had increased, as well as their level of resistance to echinocandins, while, despite the reduction in the number of *C. albicans* isolates, an upward trend in azole resistance was observed [20]. The primary mechanism of antimycotic resistance in *Candida* spp. is portrayed by a reduction in the effective intracellular concentration of the drug, achieved through overexpression of antimycotic efflux pumps, retention of antifungal molecules in an extracellular polysaccharide matrix from a biofilm that traps up to 70% of the total concentration of the drug, or changes in the target’s molecular structure, more precisely, substitutions of amino acids in key enzymes that are inhibited by the drug, to name one example. This complex and dynamic phenomenon pushed the research towards new ways of combating resistant fungi, from new classes of antifungal drugs to antibodies, plant metabolites and, with promising results, even probiotics [21].

A strong cause for concern is the presence of *Candidozyma auris*, ranked fifth among the candidoses studied, found particularly in samples between 2022 and 2024. This emerging species follows an alarming upward trend, in both the United States and Europe [22], and exhibits an alarming fluconazole resistance rate in our retrospective study, consistent with the WHO’s estimation of 87–100%. The pathogen is associated with a longer ICU hospitalization and more frequent urinary catheterization, even though mortality rates are comparable with non-*Candidozyma auris* infections [23].

As mentioned before, the majority of our isolates came from urine samples, where *Candida* species were almost exclusive, with the exception of 1% of samples positive with another fungus, namely *Trichosporon asahii*. The second most common sample was sputum, whereas the lowest numbers of samples was represented by synovial fluids and inguinal swabs. When it comes to respiratory system infections, the most common fungal pathogen found in sputum and bronchial aspirates was *Candida albicans*, but in tracheal aspirates, another fungal genus is observed to be the most frequent, represented by *Aspergillus flavus*, the most common species that causes invasive infections [24]. *Aspergillus* species were also identified in diverse secretions, consistent with the literature reports [25] showing that infections are most frequently caused by *A. fumigatus*, *A. flavus*, *A. niger* and *A. terreus*, none of which were detected in urine cultures. In stool cultures, fungi are typically transient and do not survive gastrointestinal conditions; thus, their detection suggests infection rather than colonization. From an alternative viewpoint, since the gastrointestinal tract is continuously exposed to oral microorganisms through swallowed saliva, the oral cavity is regarded as the primary source of *Candida albicans* detected in stool [26]. Moreover, the prevalence of the species is higher in Western populations, and it may be linked to higher consumption of carbohydrates [27,28]. Our results showed, however, that most positive stool samples involved *C. glabrata*, closely followed by *C. albicans*. Analysis of cerebrospinal fluid samples revealed that the most frequent fungal pathogen was *Cryptococcus neoformans*, an opportunistic and invasive fungal pathogen commonly linked to diabetes, renal and hepatic chronic disorders and HIV infections, with approximately 1 million cases and 625.000 attributable deaths reported annually [29]. Just one cerebrospinal fluid sample was positive with *Candida albicans*, a species rarely detected in this type of sample [30]. Most of the systemic fungal infections were caused by *Candida* species, among which *Candida parapsilosis* ranked first, followed by the newly emerging *Candidozyma auris*.

As for *Aspergillus* species, *A. flavus*, *A. fumigatus* and *A. niger* emerge as the most frequently encountered pathogens, which we anticipated from previous results when we looked at fungal distribution in various samples. Azole resistance is, furthermore, particularly problematic when it comes to these critical fungal pathogens, especially *Aspergillus fumigatus*. Fluconazole resistance rates have risen for filamentous fungi, and as a result, the use of amphotericin B has increased, with considerable impact [31]. Amphotericin B works by targeting ergosterol in the cell membrane, and so, resistance to polyenes occurs mainly through the use of other sterols in the cell membrane [21]. Our study found that the strains were most resistant to azoles and amphotericin B. This is of major concern, especially when looking at the results regarding the outcome of the patients admitted to the ICU, where more than half of the patients who suffered from an infection associated with these three main species did not survive.

Two species of the *Cryptococcus* genus are medically important, namely *Cryptococcus neoformans* and *Cryptococcus gattii.* The first one, *C. neoformans*, is typically isolated in immunocompromised patients, while *C. gattii* affects immunocompetent patients and has a mortality rate between 13 and 33% [29]. However, in the last decade, the incidence of *C. neoformans* infections has been reduced due to efficient antiviral treatment against HIV [32]. *Cryptococcus neoformans* is the only species found in our studied samples, mainly in cerebrospinal fluid and blood, but also from one wound sample and one sputum sample. Resistance to fluconazole was also observed for a small number of strains. Treatment can be complicated by the fact that this fungal agent exhibits intrinsic resistance to echinocandins, and multidrug resistance is fairly common, as this invasive pathogen is generally acquired from environmental reservoirs, although resistance is not typically accumulated over time, as occurs with other pathogens, due to it not being transmitted from one patient to another or from a patient back to environmental reservoirs. Nonetheless, the rise in fluconazole resistance is real and can be explained by agricultural drug exposure, and thus, it is of major concern and must be closely monitored [33].

Among the fungal species identified in our laboratory database marked down as “other” and recognized by the WHO as high-risk pathogens are *Fusarium* spp., *Rhizopus* spp. and *Mucor* spp. These fungi often display elevated minimum inhibitory concentrations, and since clinical breakpoints for susceptibility and resistance have not yet been defined, management of invasive infections caused by these fungi remains especially challenging.

## 5. Conclusions

In conclusion, this study provides an invaluable bird’s-eye view of the current situation regarding fungal infections, which are on the rise, as it spans an 8-year period, confirming that *Candida* species remains the most frequently isolated fungi, even in the ICU. *Candida albicans* isolates are starting to develop antifungal resistance, as only a small percentage was susceptible to all antifungals tested, with the highest rates of resistance observed for micafungin, amphotericin B and fluconazole, whereas *C. krusei* and *C. parapsilosis* exhibited the highest rates of resistance to fluconazole. The emerging and intimidating *Candidozyma auris* is slowly making its way into hospitals, bringing devastating fluconazole resistance. High azole resistance rates were also reported for *Aspergillus fumigatus*, as well as amphotericin B, illustrating a high ICU mortality rate, alongside other members of the genus, namely *A. flavus* and *A. niger.*

We strive to raise awareness of this often-overlooked type of pathogen and spread a major key lesson: antifungal resistance is not to be treated lightly, as it is still a complex and dynamic threat, with devastating consequences. More studies are needed to further deepen the understanding we have of familiar fungal pathogens, as well as to uncover crucial information on newly emerging fungi (for example, *Candidozyma auris*). Research on antifungal resistance trends should be continuously performed, ideally in diverse geographical areas, for us to be able to keep up with and combat this global health issue and justify funding further research into new ways to combat antifungal resistance, for example, antibodies and probiotics, antibiofilm agents and antifungal vaccines, as it is highly necessary in the context of increasing diversity of fungal species, infection patterns and resistance mechanisms.

## Data Availability

Data set available on request from the authors.

## Abbreviations

The following abbreviations are used in this manuscript:

MALDI-TOF MS	Matrix-Assisted Laser Desorption Ionization Time-of-Flight Mass Spectrometry
WHO	World Health Organization
ICU	Intensive Care Unit
HIV	Human Immunodeficiency Virus
